# Phenotypic plasticity and secretory heterogeneity in subpopulations derived from single cancer cell

**DOI:** 10.1016/j.apsb.2025.02.039

**Published:** 2025-03-15

**Authors:** Zhun Lin, Siping Liang, Zhe Pu, Zhengyu Zou, Luxuan He, Christopher J. Lyon, Yuanqing Zhang, Tony Y. Hu, Minhao Wu

**Affiliations:** a School of Pharmaceutical Sciences, Sun Yat-sen University, Guangzhou 510006, China; b Zhongshan School of Medicine, Sun Yat-sen University, Guangzhou 510080, China; c Center of Cellular and Molecular Diagnosis, Tulane University School of Medicine, New Orleans, LA 70112, USA

**Keywords:** Single-cell analysis, Microfluidic, Cancer stem cells, Phenotypic transition, Phenotypic plasticity, Tumor heterogeneity, Autocrine secretion, Growth factor

## Abstract

Single-cell analysis of phenotypic plasticity could improve the development of more effective therapeutics. Still, the development of tools to measure single-cell heterogeneity has lagged due to difficulties in manipulating and culturing single cells. Here, we describe a single-cell culture and phenotyping platform that employs a starburst microfluidic network and automatic liquid handling system to capture single cells for long-term culture and multi-dimensional analysis and quantify their clonal properties *via* their surface biomarker and secreted cytokine/growth factor profiles. Studies performed on this platform found that cells derived from single-cell cultures maintained phenotypic equilibria similar to their parental populations. Single-cell cultures exposed to chemotherapeutic drugs stochastically disrupted this balance to favor stem-like cells. They had enhanced expression of mRNAs and secreted factors associated with cell signaling, survival, and differentiation. This single-cell analysis approach can be extended to analyze more complex phenotypes and screen responses to therapeutic targets.

## Graphical abstract

A starburst microfluidic chip was designed for achieving single-cell capture, proliferation, secretion, staining, and recovery to study the phenotypic equilibria among single-cell derived subpopulations.

**Figure:**
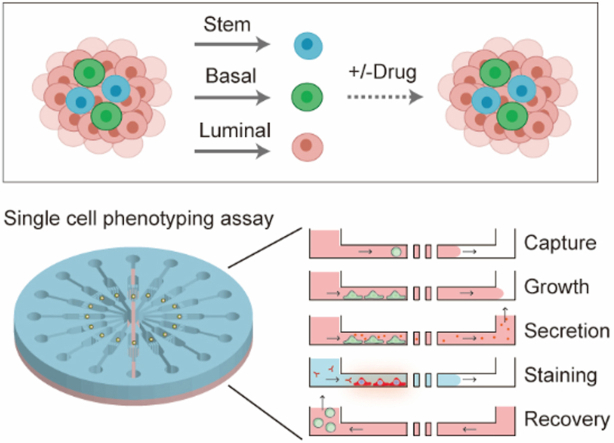

## 1 Introduction

Phenotypic plasticity influences cancer initiation, progression, and therapy resistance^1^, and thus, regulation of cellular decision-making and state transitions is a crucial factor in tumor development^2^. A better understanding of the coexistence and evolution of distinct cancer cell states within a tumor is of fundamental interest as it can potentially facilitate the development of more efficacious therapies^3^^,^^4^. However, the study of phenotypic equilibriums in cell populations disregards the heterogeneity of individual cells^5^^,^^6^, as evidence from epigenomic and genomic sequences suggests heterogeneity within distinct cell types^7^^,^^8^. Analysis of subpopulations that derive from single cells could provide valuable insight into the dynamics of tumor states.

The balance between cell renewal and differentiation is likely regulated by intrinsic and extrinsic factors^9^, particularly growth factors, glycoproteins, and cytokines^10^, which are essential in coordinating growth signal responses during development^10^^,^^11^. Cancer cell-secreted molecules can also affect chemoresistance in an autocrine manner^12^. Cytokines and cytokine receptors have been extensively investigated as targets for cancer therapeutics or treatments and have prompted clinical trials of several cytokines or cytokine antagonists^13^^,^^14^. However, analyzing a cytokine’s role during tumor state transitions is complicated by the low abundance of these factors, their varied effects, and interactions and redundancies with other members of the overall cytokine milieu^15^. Multi-dimensional characterization of single cells, including evaluation of their secretory heterogeneity, would be a promising approach for observing and analyzing phenotypic plasticity.

Early single-cell analysis methods primarily relied on manually picking under the microscope^16^^,^^17^ or flow cytometry sorting^18^^,^^19^. Still, the former is labor-intensive and inefficient, while the latter is complex, expensive, and limited to fixed-time points^20^. Microfluidics has recently emerged as an ideal tool for efficient single-cell studies. However, hydrodynamic capture chips can cause cell injury, have microscale limits that can challenge long-term cell culture, and make it difficult to avoid interferences among captured cells^21^, ^22^, ^23^, ^24^, ^25^. Most methods that analyze single-cell secretion properties rely on functionalized surfaces or beads close to individual cells^26^, ^27^, ^28^, ^29^. However, surface area can reduce the sensitivity, capture capacity, and number of factors analyzed by such approaches, and their multiple manipulations can also damage the analyzed cells and their culture environments^30^. It is, therefore, challenging to simultaneously detect secreted molecules in single-cell cultures at high-throughput^8^^,^^21^^,^^31^. However, new microfluidic designs that integrate all required analysis processes onto a single device should permit comprehensive single-cell profiling studies^32^.

Here, we present a streamlined analysis method that employs small sample volumes to perform single-cell phenotyping. This approach employs a microfluidic chip with a starburst channel network to automate liquid handling *via* a liquid-transfer workstation, which can simultaneously profile cell phenotypes and isolate cell/conditioned media for further characterization. In this study, we employed a panel of breast cancer cell lines to analyze this device’s single-cell segregation and culture characteristics. We found that it could efficiently produce and maintain long-term cultures that could be serially analyzed on-chip to evaluate their surface biomarker and cytokine secretion profiles. Single-cell cultures performed on this chip recapitulated the phenotypic composition of their source populations when analyzed by on-chip immunofluorescence and by off-chip flow cytometry studies and exhibited phenotypic plasticity and secretory heterogeneity that was affected by treatment with chemotherapeutics. Further, cytokine and RNA analyses identified factors enriched during these single-cell cultures' proliferation and phenotypic transition. These findings thus provide evidence for the utility of this on-chip single-cell culture approach as an efficient means to screen therapeutic targets and profile complex phenotypic traits in single-cell cultures to capture the diversity of responses that may occur in the total population.

## 2 Materials and methods

### 2.1 Cell culture

Breast cancer cell lines were obtained from ATCC (USA). G12V cells were a kind gift from Prof. Xiaolei Zhang’s laboratory (Sun Yat-sen University). SUM159 and 4T1 cell lines were cultured in RPMI 1640 medium (Gibco; ThermoFisher Science) with 10% (*vol*/*vol*) fetal bovine serum (Gibco; ThermoFisher Science). MDA-MB-231, MDA-MB-468, and MCF-7 cell lines were cultured in DMEM medium (Gibco; Thermo Fisher Science) with 10% FBS. The SKBR3 cell line was cultured in McCoy’s 5A medium (Gibco; Thermo Fisher Science) with 10% FBS.

### 2.2 Chip design and fabrication

The photo mask was designed using AutoCAD (Autodesk 2020, USA), and the silicon molding master was fabricated using soft photolithography on 4-inch wafers. The process included spin-coating (3000 rpm, 30 s) of SU8 3025 negative photoresist (MicroChem Corp, USA), soft-baking (2 min at 65 °C; 12 min at 95 °C), UV-exposing (150 mJ/cm^2^), postexposure-baking (2 min at 65 °C; 3 min at 95 °C), and developing (5 min, SU-8 developer, MicroChem Corp, USA). The mixture of poly(dimethylsiloxane) (PDMS) Sylgard 184 monomer base and curing agent (Dow Corning, USA) in a 20:1 ratio was poured into the silicon mold and baked at 80 °C for 2 h, then the PDMS layer was peeled away from the mold, holes were punched in it, and it was bonded to the glass layer by plasma treatment.

### 2.3 Cell loading

Chips were loaded with 20 μL of cells resuspended in Ham’s F12 medium (Gibco; ThermoFisher Science) supplemented with 5% calf serum, insulin (5 μg/mL, Beyotime), and hydrocortisone (1 μg/mL, Stemcell) by micropiette addition to the central inlet followed by 40 μL cell culture media to disperse cells to the 120 radial culture chambers. Residual cells remaining in the inlet were removed, and the chip was slowly perfused with culture media.

### 2.4 Single-cell proliferation assay

After cell loading, culture media with or without different drug concentrations was added to the chip. Cell images were captured with a high-resolution camera connected to an inverted optical microscope (Olympus, X81, Japan). The number of cells that grew from each clone was recorded each day, and cells were stained after 9 days of culture by adding Calcein/AM solution (1:1000 dilution, Beyotime, China) to the chip inlet.

### 2.5 Cell retrieval

Chip channels were first washed by introducing 20 μL PBS to the outlet well to generate negative pressure, after which the chip was perfused with 10 μL 0.25% trypsin for 2 min to release cultured cells and cause the flow towards the inlet well, where they were recovered and processed for further analyses.

### 2.6 Fluorescence-activated cell sorting

Cells were trypsinized, counted, washed with PBS, and incubated for 30 min at room temperature with fluorescently tagged antibodies specific for human cell-surface markers, including PE-labeled anti-EpCAM (1.25 μg/mL, eBioscience™, 12-5791-82, USA), PE-Cyanine7-labeled anti-CD24 (2.5 μg/mL eBioscience™, 25-0242-82, USA), and APC-labeled anti-CD44 (0.6 μg/mL, eBioscience™, 17-0441-82, USA). Cells were then washed to remove unbound antibodies and analyzed by flow cytometry (Beckman Coulter, CytoFLEX S, USA) within 1 h after staining. Cell subpopulations used for subsequent culture were segregated using a cell sorter (Beckman Coulter, MoFlo Astrios EQs, USA).

### 2.7 Immunology fluorescence analysis

Cell nuclei were stained by adding 5 μL of Hoechst 33258 (0.1 mg/mL) to ∼50 μL of media that was then added to the central inlet well to perfuse the radial culture chambers under a constant flow rate that was maintained for 10–15 min, and followed by rinsing the chip with culture media. Chips were perfused with PE-conjugated anti-EpCAM (1.25 μg/mL, eBioscience™, 12-5791-82, USA), FITC-conjugated anti-CD24 (2.5 μg/mL eBioscience™, 11-0247-41, USA), APC-conjugated anti-CD44 (0.6 μg/mL, eBioscience™, 17-0441-82, USA) antibodies to stain these surface markers, and then imaged using a FV3000 confocal microscope (Olympus, Japan).

### 2.8 Conditioned media collection

Cells were washed with PBS and incubated in serum-free medium for 48 h, after which conditioned media was collected from each outlet well, centrifuged at 875×*g* (Eppendorf, D-16C, Germany) at RT for 10 min to remove cell debris, and then aliquoted and stored at −20 °C before use.

### 2.9 Secretome analysis

Concentrated conditioned media (200 μg total protein) was mixed with blocking buffer and incubated with human growth factor (Abcam, 134002, England) and cytokine (Abcam, 193656, England) antibody array membranes at 4 °C overnight using the manufacturer’s protocols and assay reagents unless otherwise specified. These membranes were then washed and incubated with a mixture of biotin-conjugated antibodies specific to all the array targets for 2 h at RT, washed, incubated with HRP-conjugated streptavidin for 2 h at RT, washed, and incubated with ECL reagents to allow bound antibodies to be visualized by autoradiography. The average signal of a pair of duplicate array spots was normalized using the negative control spots as a background value, and relative intensities were determined by comparing signals detected for each factor in the two groups by Image J software.

### 2.10 CCK8 assay

100 μL volumes containing 1000 cells were added to the wells in 96-well plates, cultured for 24 h, and then supplemented with 100 μL volumes containing varying concentrations of paclitaxel (Sigma, USA), 5-fluorouracil (Sigma, USA), or DMSO (negative control). Cells were then cultured for 6 days and then incubated for 2 h with CCK8 (Biosharp, China) reagent (100 μL/mL medium) and the absorbance of each sample well was read at 450 nm using a microplate reader (BioTek, Synergy H1, USA). Inhibitory concentrations were calculated by nonlinear regression analysis using GraphPad Prism software (version 9.1.0, GraphPad, Bethesda, MD, USA).

### 2.11 RNA-sequencing analysis

Total RNA was extracted using a Trizol reagent kit (Invitrogen, Carlsbad, CA, USA) according to the manufacturer’s protocol. RNA quality was assessed on an Agilent 2100 Bioanalyzer (Agilent Technologies, Palo Alto, CA, USA) and checked using RNase-free agarose gel electrophoresis. After total RNA was extracted, eukaryotic mRNA was enriched by Oligo (dT) beads. Then, the enriched mRNA was fragmented into short fragments using fragmentation buffer and reversely transcribed into cDNA by using NEBNext Ultra RNA Library Prep Kit for Illumina (NEB #7530, New England Biolabs, Ipswich, MA, USA). The purified double-stranded cDNA fragments were end-repaired, and A base was added and ligated to Illumina sequencing adapters. The ligation reaction was purified with the AMPure XP Beads (1.0×). Moreover, polymerase chain reaction (PCR) is amplified. The resulting cDNA library was sequenced using Illumina Novaseq6000 by Gene Denovo Biotechnology Co. (Guangzhou, China).

### 2.12 Quantitative PCR

Total cellular RNA was extracted with Trizol reagent and 2 μg total RNA was reverse-transcribed by using a Hifair II 1st-strand cDNA Synthesis Kit (YEASEN, Shanghai, China). Quantitative real-time PCR analyses were performed in a 10 μL reaction containing SYBR Green Master Mix (Vazyme) by using the CFX96 Real-Time PCR System (Bio-Rad, USA). The amplification of target mRNA sequences was normalized against the signal produced by a *β*-actin target amplicon. Specific primer sequences are listed in Supporting Information Table S1.

### 2.13 Luciferase reporter assay

The experiment was conducted using a Luciferase labeling kit (FUHeng Biology, Shanghai, China). Specifically, when SUM-159 cells grew to ∼30% confluence in 6-well plates, the culture medium was refreshed. Polybrene (final concentration: 10 μg/mL) and 20 μL Luc labeling solution were added to the plate respectively and mixed together. After 48 h, puromycin (3–5 μg/mL) was added to the culture medium for 48 h. Finally, a dual-luciferase reporter assay system (Promega, Beijing, China) was used to detect the effect of luciferase labeling.

### 2.14 Animal studies

All animal experiments were approved by the Ethics Committee Board for Human and Animal Experiments at Zhongshan School of Medicine of Sun Yat-sen University (SYSU-IACUC-2023-B0109). Wild-type luc-SUM159 or luc-SUM159 (G12V) cells modified to overexpress the activated KRAS G12V mutant (10^5^ cells) were suspended in 100 μL PBS and mixed with an equal volume of Matrigel (Corning, USA) supplemented with or without EGF (10 μg/g) and injected into the fourth mammary fat pad of 8- to 12-week-old female Balb/c Nude mice (19–25 g) that were randomly assigned to each experimental group. At the indicated time points after injection, these mice were intraperitoneally injected with luciferin (150 mg/kg, Aladdin, USA). Mice were then returned to their cages for 5 min to permit biodistribution, and then anesthetized with 2% isoflurane gas and imaged at 5 min intervals to detect photon emission (NightOWL II LB983, Germany). Total flux (cps) and area (mm^2^) values were calculated from this data and corrected for tissue depth.

### 2.15 Statistical analyses

Prism GraphPad software was used to analyze and graph the presented data, and graphs depict mean ± standard deviation (SD) results of biological replicates unless otherwise specified in the text.

## 3 Results

### 3.1 Automated microfluidic assay design and operation

**Figure 1:**
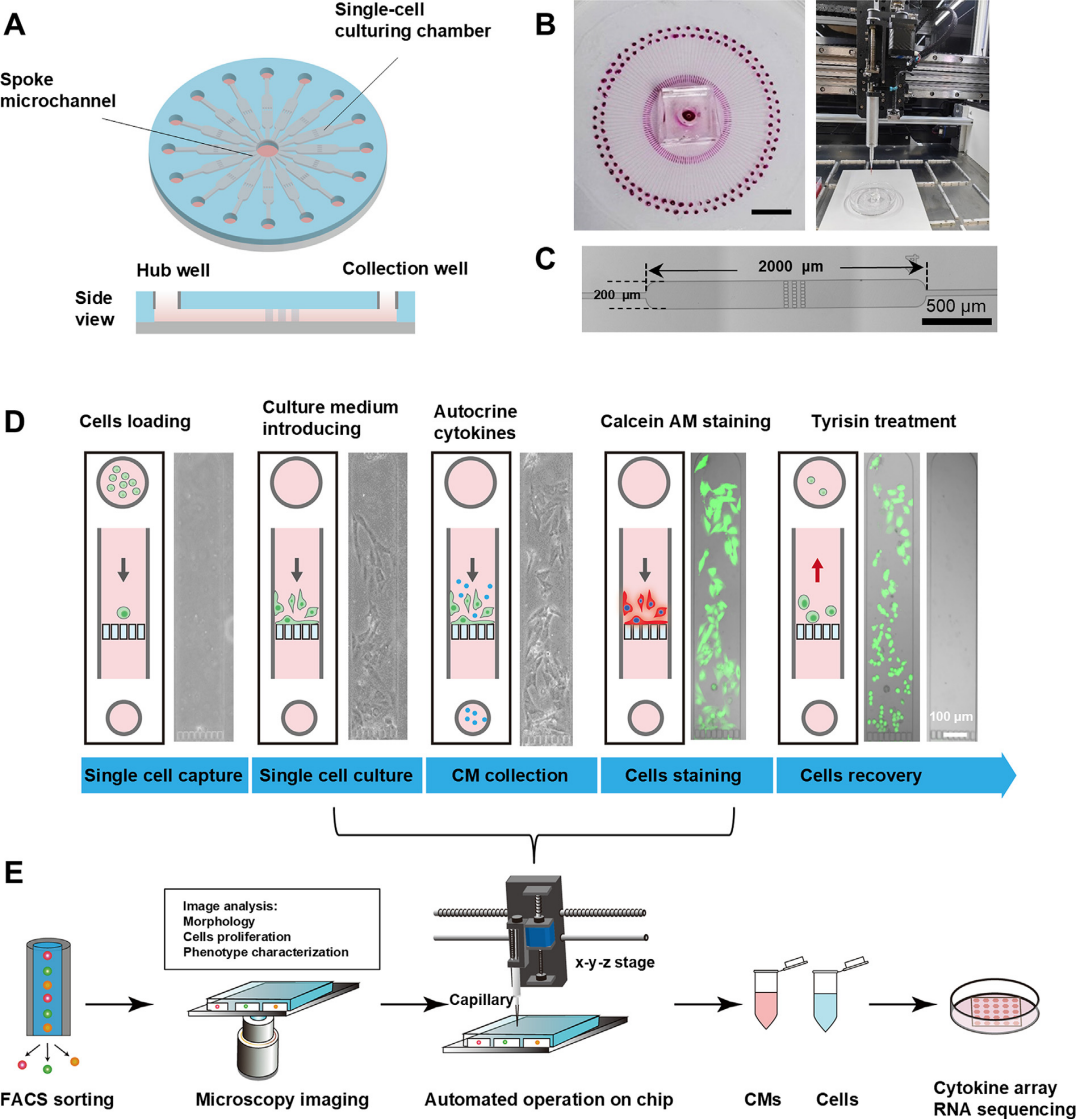
Single-cell phenotyping assay and protocol. (A) Schematic of the starburst-shaped chip design for single-cell heterogeneity analysis features a central hub well with radial microchannels connected to individual cell capture and culture chambers that terminate in collection wells. (B) Images of the assembled microchip with 120 culture chambers after dye loading (Scale bar = 1 cm) and on its liquid transfer platform. (C) Images of an individual cell culture chamber with its three parallel rows of micro barriers designed to restrict cell passage. Scale bar = 500 μm. (D) Schematic of the chip loading, cell culture, conditioned media (CM) capture, cell staining, and cell recovery procedures. Scale bar = 100 μm. (E) Schematic of the setup of single-cell cultures of specific cell subtypes, image analyses, and liquid handling procedures for supernatants and cell collections required for cytokine and RNA analyses.

To facilitate the robust clonal expansion of single cells that exhibit specific phenotypes in a confined and controlled environment, we designed a starburst-shaped device that employs a hub-and-spoke network to enable single-cell capture and culture (Fig. 1A). In this method, dilute cell populations are loaded into the central hub of a well 5 cm diameter chip, allowing spoke microchannels that originate at this hub and terminate in separate collection wells to uniformly distribute cells into 120 distinct single-cell capture chambers employed for cell culture and observation (Fig. 1B). However, the number of capture chambers can be increased by increasing the diameter of the chip. Each chamber contains three sets of parallel micro barriers formed by square pillars with 4 μm gaps that confine input cells within a 200 μm wide by 1000 μm long chamber while accommodating the size heterogeneity of single-cell populations and the flow of conditioned media (CM) through these chambers (Fig. 1C). Each microchamber has a capacity of 300–2000 cells ranging from 10 to 25 μm in diameter. This chip requires only 20 μL, significantly reducing sample and reagent consumption, and its design prevents intercellular interference, distinguishing it from existing single-cell separation structures^33^. This microchip is mounted on a liquid-transfer workstation that employs a syringe pump connected to a pipet tip to achieve precise control of all liquid manipulation steps. Software control of *x*-*y*-*z* stage movement permits specific volumes to be deposited into or aspirated from designated reservoirs within the microchip with 0.1 μL precision, 0.02 mm resolution, and a linear speed range of 4–500 mm/s within its 450 (*L*) × 350 (*W*) × 150 (*H*) mm movement range.

We first evaluated the ability of this chip-liquid handler system to automate phenotype analysis and retrieve cells and CM for experiments. After programming the *x*-*y*-*z* stations and pipetting procedures for the liquid handler, this integrated device could automatically load: (1) cells into the hub well to distribute single cells into the radial microchambers; (2) culture medium into these microchambers to permit the growth of subpopulations from single cells; (3) culture medium to collect autocrine cytokines from these subpopulations; (4) a fluorescence dye that permitted visual characterization of subpopulation phenotypes; and (5) trypsin into the collection well to detached these cells for retrieval at the hub (Fig. 1D). Single cell-derived subpopulations from individual microchambers were subjected to microscopic imaging analyses to identify cell morphology, proliferation, and phenotypic changes over 9 days of culture, these cells were then subsequently analyzed by flow cytometry to identify specific cell phenotypes through the detection of specific biomarkers and by RNA sequencing to identify gene enrichment patterns, while collected CM was analyzed by high-throughput cytokine arrays (Fig. 1E).

### 3.2 Quantification of single-cell capture, proliferation, and secretion of growth factors

**Figure 2:**
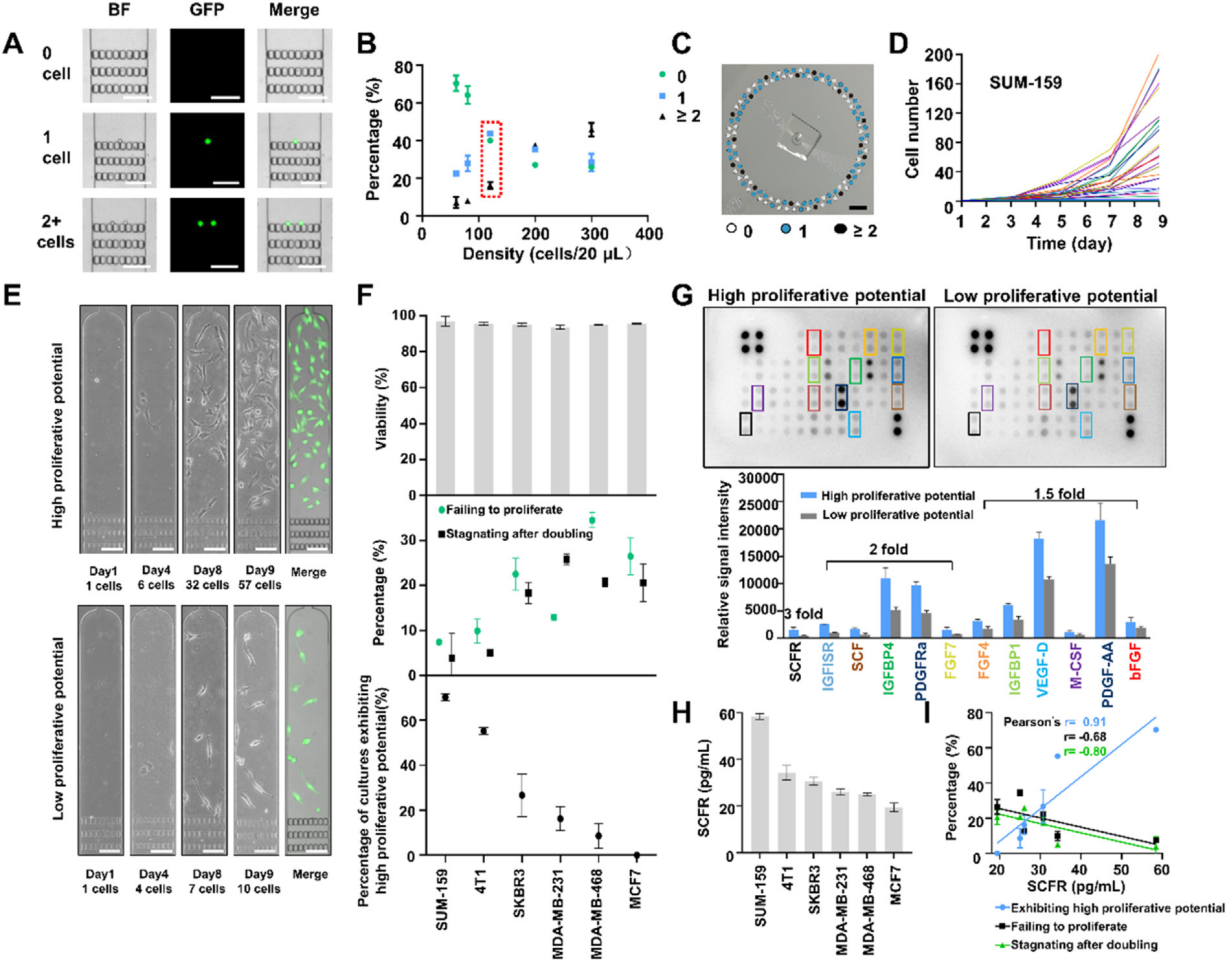
Single cells capture and proliferative heterogeneity. (A) Fluorescence images distinguish chambers containing 0, 1, or 2+ cells. Scale bar = 100 μm. (B) Single-cell capture efficiency at different cell concentrations (*n* = 3/group), where a red rectangle indicates the highest single-cell capture rate and (C) the on-chip distribution of capture events. Scale bar = 5 mm. (D) SUM-159 single-cell culture growth rate distribution. (E) Images of unstained and calcein-AM-stained MDA-MB-231 cell cultures exhibiting high and low proliferative potential. Scale bar = 100 μm. (F) Single-cell culture viabilities at 24 h (top), and the percentage of these cultures that do not proliferate or stagnate after doubling (middle) or exhibit high proliferative potential after 9 days culture for six breast cancer cells (*n* = 3/each). (G) Relative growth factor expression in CMs of SUM-159 cells with high and low proliferative potential, where colored boxes indicate factors with differential array signal, which is graphed as the mean and standard deviation of the densitometric values of paired array spots. (H) SCFR levels in CMs of six breast cancer cell lines. (I) Correlation of SCFR concentration *versus* the percentage of single-cell culture cells that do not proliferate, stagnate after one division or exhibit high proliferation, indicating the best-fit line and Pearson’s correlation coefficient for each comparison.

Single-cell capture rates for this chip were predicated on the stochastic allocation of dilute cell samples into its 120 radial capture chambers. To determine the optimal cell concentration for single-cell capture, the proportion of these chambers containing no cells, single cells, or two or more cells was analyzed when these chips were loaded with samples with different cell densities. This analysis found that the highest single-cell rate (44%) was achieved when the chip was loaded with 20 μL of a 6000 cell/mL sample to achieve a loading sample containing 120 cells (Fig. 2A and B). The radial distribution of wells containing single cells was relatively uniform (Fig. 2C) and no difference in single-cell capture efficiency was detected when chips were loaded using manual and automated processes (Supporting Information Fig. S1A). Cells captured on the chip had the same size distribution as the input sample, indicating that cell size and flow-induced shear stress did not influence the captured cell size distribution (Fig. S1B). Single cells stayed in front of the microcarriers and adhered *in situ*. Subsequently, cells migrated and grew toward the direction of medium perfusion and eventually occupied the whole culture chamber. SUM-159 cell proliferation was analyzed by dye staining until culture day nine, when the analysis was ended to prevent chamber overgrowth by the rapidly proliferating cells (Fig. S1C). However, extended culture times could be possible when analyzing more slowly proliferating cells.

Single-cell isolates from clonal cell populations revealed proliferative heterogeneity when captured and cultured on this chip (*e.g.*, some failed to proliferate or stagnated after a single cell division) (Fig. S1D and S1E), indicating that such single-cell cultures could detect variations that are potentially masked by interactions in bulk cultures (Fig. 2D and Fig. S1F and S1G). In this analysis, SUM-159, MDA-MD-231, and MCF-7 cells revealed high, intermediate, and low single-cell growth rates. Single-cell isolates from different populations were categorized as having high or low proliferative potential if they respectively contained more than or less than 16 cells after 9-days culture (median cell number in MDA-MB-231 single-cell cultures at this interval) (Fig. 2E). Six breast cell lines (SUM-159, 4T1, SKBR3, MDA-MB-231, MDA-MB-468, MCF7) exhibited consistent viability 24 h of on-chip culture but varying rates of proliferation failure and highly variable proliferative potential (Fig. 2F).

Previous studies have reported that autocrine growth factors secreted by breast cancer cells play a crucial role in their *in vitro* proliferation^34^. To evaluate whether differential growth factor secretion was associated with the differential proliferative capacity of single-cell isolates, we collected CM from wells containing subpopulations exhibiting high and low proliferation potential. We analyzed whether these CM samples revealed disparities in growth factor secretion after normalization for protein content. This analysis found that 12 such proteins had at least 1.5-fold greater expression in CM of cells with high proliferative potential (Fig. 2G). Stem cell factor receptor (SCFR), insulin-like growth factor-I soluble receptor (IGF Ⅰ-SR), stem cell factor (SCF), insulin-like growth factor binding protein 4 (IGFBP4), platelet-derived growth factor receptor alpha (PDGFR*α*), fibroblast growth factor (FGF7) exhibited the most notable (>2-fold) increases in CM of highly proliferative cells, suggesting that they may play essential roles in mediating the proliferative capacity of single-cell cultures. SCFR revealed the most significant increase (>3-fold) among these factors, and high SCFR levels were associated only with cells that had high single-cell proliferative capacities, suggesting that SCFR plays a crucial role in mediating proliferation in breast cancer cells (Fig. 2H and I).

### 3.3 Phenotypic equilibrium in single cells-derived subpopulations

**Figure 3:**
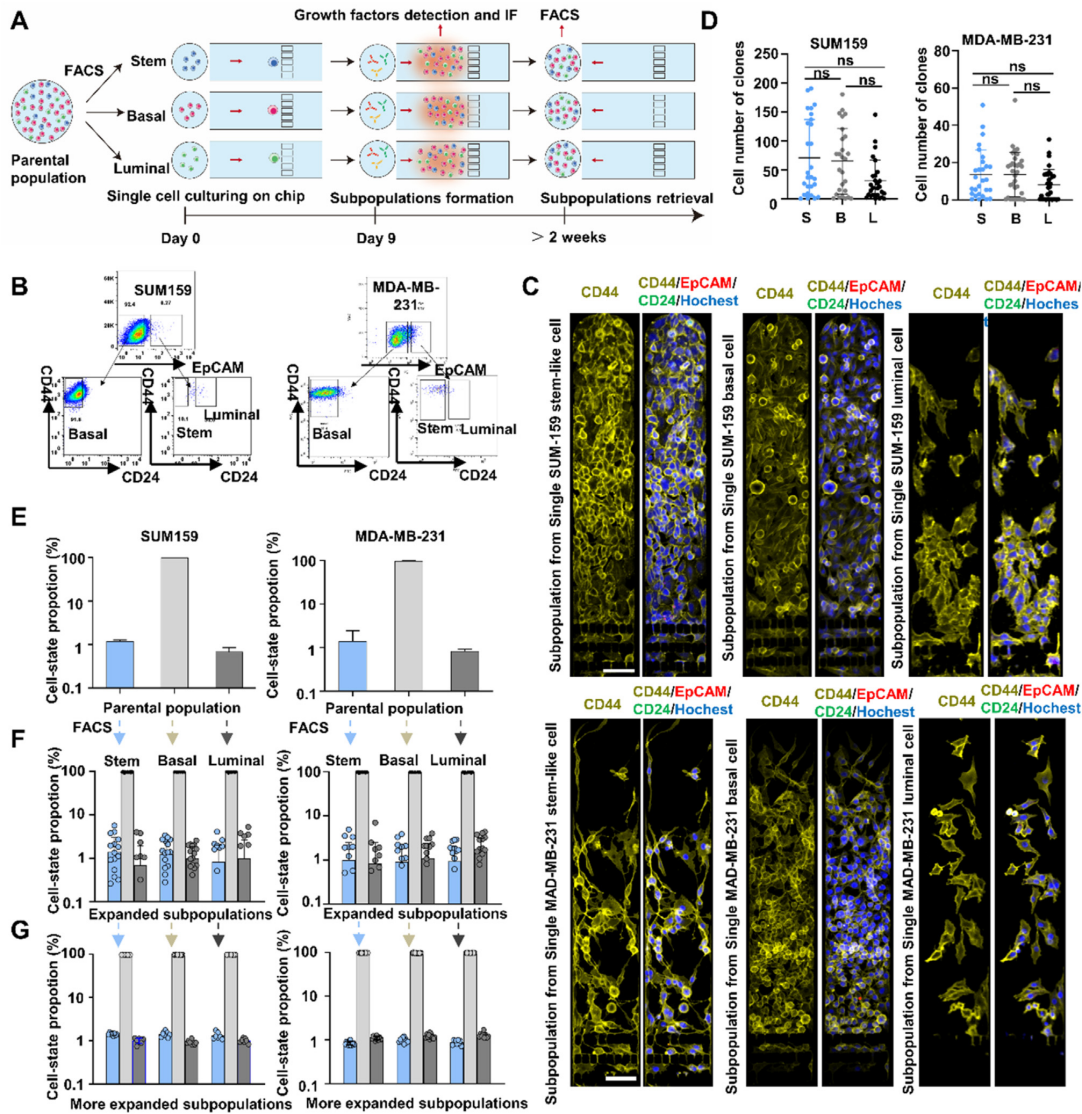
Cells arising from single-cell cultures interconvert between phenotypic states. (A) Schematic of the protocol employed to analyze cell–state transition. (B) Flow cytometry gating strategy used to isolate stem-like (CD44^high^CD24^neg^EpCAM^high^), basal (CD44^high^CD24^neg/low^EpCAM^low^), and luminal (CD44^high^CD24^high^EpCAM^high^) cells from SUM159 and MDA-MB-231 cell cultures. (C) Cell numbers in clones derived from three cell subtypes after 9 days of on-chip culture. Data was analyzed by one-way ANOVA with Tukey’s multiple comparison test (ns, not significant). Scale bar = 100 μm. (D) Confocal micrographs of subpopulations derived from single stem, basal, and luminal cells after immunostaining for CD44, CD24, and EpCAM (scale bar: 100 μm. (F, G) Bar charts indicating cell state distributions, as determined by fluorescence staining following (F) 9 days on-chip culture (*n* > 50 cells/clone analyzed by visual inspection) and (G) 2–4 weeks off-chip culture (*n* > 1000 cells analyzed by FACS).

Next, we utilized this platform to conduct single-cell phenotypic analyses. Breast cancers are reported to contain three mammary epithelial cell states defined by their surface markers: stem-like (CD44^high^CD24^neg^EpCAM^high^), basal (CD44^high^CD24^neg/low^EpCAM^low^), and luminal (CD44^high^CD24^high^EpCAM^high^) cells^35^^,^^36^, which remain constant through multiple divisions in culture^2^. To investigate if phenotypic equilibrium persists in single-cell cultures, cells belonging to these differentiation states were isolated by fluorescence-activated cell sorting (FACS) and expanded from single-cell cultures on the chip to evaluate the persistence of their initial phenotypic state (Fig. 3A).

SUM159 and MDA-MB-231 cells exhibited similar cell state distributions, with most isolated cells exhibiting basal (B) differentiation and few displaying stem-like (S) or luminal (L) phenotypes (Fig. 3B). B, S, and L percentages were similar in bulk SUM-159 and MDA-MB-231 cell cultures (98.1%, 1.19%, and 0.64% *versus* 97.8%, 1.41%, and 0.81%). These cell lines' FACS-isolated B, S and L fractions were then used to generate single-cell on-chip cultures. Single SUM159 or MDA-MB-231 cells of the same phenotype exhibited varying proliferative potential; however, mean proliferative potential did not differ among these three phenotypes (Fig. 3C). Next, these cultures were stained with a cell-permeable nuclear stain and fluorescent antibodies specific for EpCAM, CD24, CD44 to determine the proportions of S/B/L cells produced by single-cell cultures of each subtype (Fig. 3D). All these cultures primarily produced B cells with very few S or L cells, similar to their parental cells (Fig. 3E and F) and similar results were obtained for bulk and single-cell cultures grown in plate wells (Supporting Information Fig. S2A and S2B). FACS analyses performed with expanded single-cell subpopulations (>10,000 cells) revealed this effect persisted with extended culture (Fig. 3G and H, Fig. S2C) and was thus due to interconversion between cell states rather than differential growth rates of B, S or L phenotype cells.

We continuously monitored B, S, and L percentages during the expansion of single SUM-159 cell cultures to analyze phenotype transition dynamics during single-cell culture. We found that single-cell S and L cultures revealed a rapid rise in the B-type cell fraction that was matched by a decrease in the initial cell phenotype without a substantial increase in the remaining cell subtype (Fig. S2D). Most new cells in single-cell B-type cultures retained a B phenotype with a low incidence of the other two cell types. Thus, proliferating cells had a low probability of transitioning to a stem-like state in both single-cell B- and L-type cultures, implying that stem-like cells can arise from non-stem-like cells during SUM-159 cell culture. Characterization of the factors/pathways that regulate this process could thus have significant implications for the understanding of breast cancer recurrence after treatment.

Next, using a growth factor array, we evaluated the cytokine secretion profile of cell populations derived from the B, S, L, and single-cell cultures. No significant differences were detected for most analyzed cytokines, except that populations derived from L-type cells exhibited higher expression of epidermal growth factor receptor (EGFR) and Insulin-like growth factor binding protein 2 (IGFBP2) than those derived from single-cell cultures of B- and S-type cells (Supporting Information Fig. S3), indicating that the secretion profiles of these mixed cell populations can be influenced by the phenotype of their parental cell.

### 3.4 Phenotypic plasticity in single-cell-derived subpopulations

Breast cancer cells can exhibit phenotypic plasticity and produce distinct subpopulations with different functionalities^2^^,^^37^. This has stimulated interest in identifying drugs that selectively target specific cell differentiation states. Next, we evaluated phenotypic transitions and secretory changes induced by selected anti-cancer drugs for specific cell states at single-cell resolution using our single-cell culture platform.

**Figure 4:**
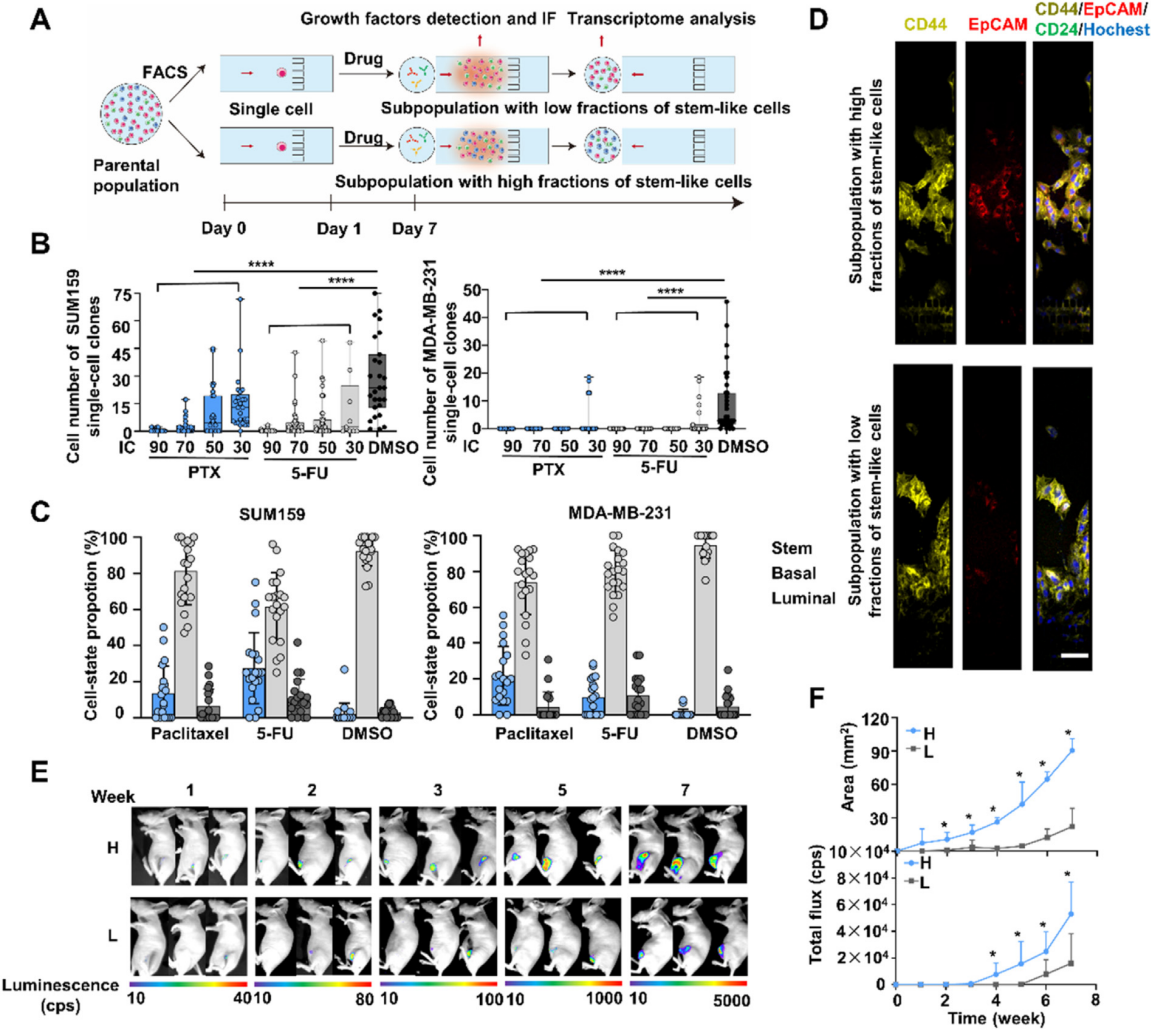
The phenotypic plasticity of single cell-derived subpopulations with treatment. (A) Schematic of the protocol used to analyze cell–state transition after drug treatment. (B) Cell numbers in clones of SUM-159 and MDA-MB-231 single-cell cultures after 7 days of culture with PTX, 5-FU, or DMSO were analyzed by one-way ANOVA with Tukey’s multiple comparison test. (∗∗∗∗*P* < 0.0001). (C) Bar charts illustrate the distribution of cells obtained from individual cells in each state with different treatments, as determined by fluorescence staining following 7 days of on-chip culture. The cell numbers of used clones were 20–50 cells. (D) Confocal images of treated subpopulations exhibiting high and low proportions of stem-like cells stained for CD44, CD24, and EpCAM expression (scale bar: 100 μm). (E) Representative bioluminescent images of tumor growth in mice injected with luc-cells with high (H) or low (L) proportion of stem-like cells. (F) Bioluminescent signal and area in mice injected with treated cell culture samples exhibiting high and low proportions of stem-like cells. Data represent mean ± standard deviations (*n* = 5 mice/group). Differences were assessed by two-tailed Mann–Whitney tests without adjustment for multiple comparisons (ns, not significant; ∗*P* < 0.05).

CCK8 assay results were first used to determine inhibitory concentrations (IC) for two commonly prescribed breast cancer chemotherapy drugs, paclitaxel (PTX) and 5-fluorouracil (5-FU), when applied to single-cell SUM-159 and MAD-MB-231 cultures (Supporting Information Fig. S4 and Supporting Information Table S2). In this analysis, single cells were cultured on-chip for 24 h and then exposed to different concentrations of PTX, 5-FU, or dimethyl sulfoxide (DMSO), which was employed as a vehicle control (Fig. 4A). SUM-159 single-cell cultures revealed variable growth rates across most of the IC range of these two drugs, MAD-MB-231 cells treated with these drugs only proliferated at the lowest tested IC value (Fig. 4B). Some single-cell SUM-159 cultures grew rapidly when treated with PTX IC_30_ or IC_50_ doses displayed growth rates similar to those observed with DMSO, as did a smaller fraction of the cultures treated with 5-FU-treated, indicating heterogeneity of drug resistance in the single cells that produced these culture populations. Notably, a small fraction of SUM-159 cultures still exhibited slow growth when exposed to IC_90_ doses of these drugs. However, only a few single-cell MDA-MB-231 cultures grew under IC_30_ conditions of either drug.

Next, we evaluated phenotypic transitions in single-cell cultures of SUM-159 and MDA-MB-231 cells after exposure to IC_70_ or IC_30_ PTX or 5-FU doses. Notably, both drug treatments increased S-type and decreased B-type mean cell percentages for both cell lines *versus* their DMSO-treated control groups (Fig. 4C), as observed in the population cell culture^2^. Furthermore, the mean stem-like cell percentages are higher than luminal cell percentages. No proliferation differences were observed in single-cell clones that had high and low percentages of stem-like cells (Supporting Information Fig. S5A) when they were segregated by the median S-type cell percentage (14.29%) of their populations across all the treatment groups (Fig. 4D). SUM-159 and MDA-MB-231 single-cell cultures differed in the proportions of single-cell clones that exhibited a high percentage of stem-like cells when treated with either PTX or 5-FU and analyzed by immunofluorescence (Fig. S5B). These results were verified by flow cytometry (Fig. S5C), which found that the high proportion of stem-like cells group had a higher percentage of S-type cells than the low proportion of stem-like cells group, consistent with the immunofluorescence data.

Since S-type cells are crucial for tumor-seeding potential^2^, we hypothesized that cells that exhibit a high proportion of stem-like cell phenotypes should have greater tumor-forming capacity than those with a low proportion of stem-like cell phenotypes. To test this hypothesis, we injected cells isolated from luciferase-expressing SUM159 single-cell clones with high and low proportion of stem-like cells into the fourth mammary fat pad of immunodeficient female BABL/c nude mice. Mice injected with the high *versus* low proportion of stem-like cells cell population revealed faster tumor formation and growth (Fig. 4E), with a four-fold increase in bioluminescent tumor area and a three-fold increase in total fluorescence intensity (Fig. 4F). Thus, the cells produced by these S-type single-cell cultures exhibit both the biomarker profile and expected *in vivo* phenotype of stem cells.

### 3.5 Secretome and transcriptome analysis of cell cultures with high stem-like cell fractions

**Figure 5:**
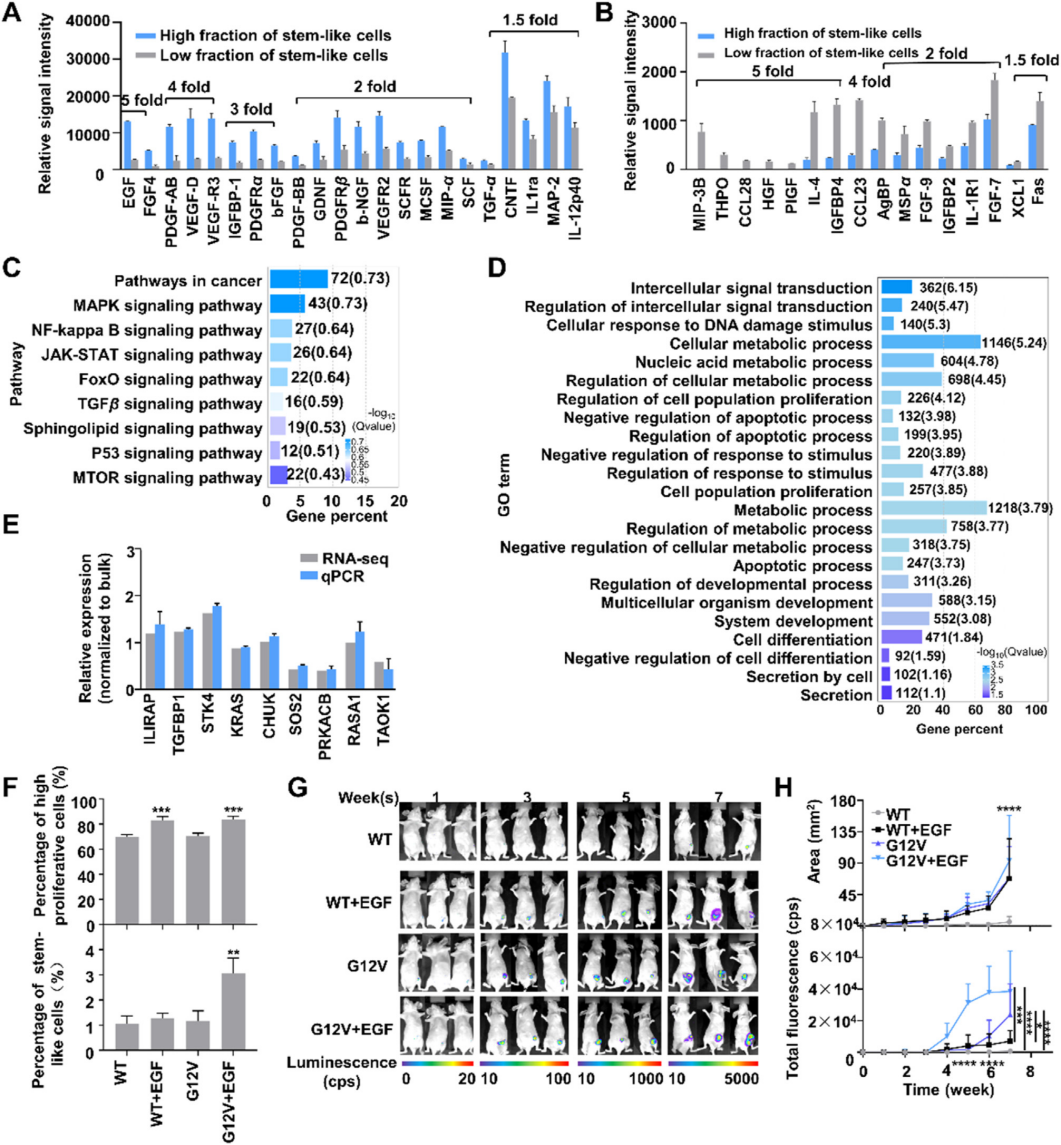
Secretome and transcriptome analysis of SUM-159 cultures exhibiting high and low percentages of stem-like cells. (A, B) Growth factors are highly and lowly expressed in CMs of cultures with high *vs*. low fractions of stem-like cells. (C) Pathway analysis and (D) gene ontology annotation of DEGs in cultures with high and low fractions of stem-like cells. (E) Validation of select DEGs identified by RNA-seq using qPCR (*n* = 3/group). (F) Percentage of cells with high proliferative capacity and stem-like phenotypes in cells derived from WT, WT + EGF, G12V, G12V + EGF single-cell cultures (*n* = 3/group), indicating differences determined by two-way ANOVA and Tukey’s multiple comparison test (∗∗∗*P* < 0.001 for EGF and G12V + EGF *vs*. WT and G12V cultures; ∗∗*P* < 0.01 for G12V + EGF *vs*. WT, WT + EGF, and G12V cultures). (G) Representative bioluminescence images and (H) quantitation of the area and total flux signal in nude mice injected with the indicated treatment groups of SUM-159 cells expressing luciferase. Data represent mean ± standard deviation values (*n* = 5 mice/group. Top: ∗∗∗∗*P* < 0.001 for G12V + EGF *vs*. all groups at 7 weeks. Bottom: ∗∗∗∗*P* < 0.0001 for WT *vs*. all groups at 5 and 6 weeks. ∗*P* < 0.05, ∗∗∗*P* < 0.001, ∗∗∗∗*P* < 0.0001 indicated comparisons at 7 weeks).

Cancer cell “stemness” phenotypes are complex traits influenced by multiple factors^38^^,^^39^, including cytokines, chemokines, and growth factors that play crucial roles in various immunological and pathophysiological processes. Growth factors and cytokines/chemokines present in the secretomes of SUM-159 cell cultures with high and low fractions of stem cells were thus analyzed using a 161-target assay (Supporting Information Fig. S6). This analysis identified 22 factors that increased >1.5-fold and 16 that decreased >1.5-fold in the samples with high-*vs.* low-stem cell subpopulations (Fig. 5A and B) that could contribute to differences in their stem-like potential. Factors demonstrating increased expression were primarily growth factors, while those with decreased expression were predominantly cytokines and chemokines. Cytokine/chemokine decreases detected in the groups with low-*versus* high stem cell percentages could have negative or neutral effects on stem cell differentiation and growth. However, several factors whose expression was increased in the groups with high-*versus* low stem cell compositions, including epidermal growth factor (EGF) and basic fibroblast growth factor (bFGF), are commonly employed as additives to stimulate or preserve stem cell phenotypes during *in vitro* cell culture^40^^,^^41^. Secretion of these factors by the stem-cell enriched samples may thus regulate their capacity for stem-cell self-renewal and maintenance. However, increased expression of SCFR in the high *versus* low-stem cell subpopulations was observed. However, no disparity in growth was observed between the groups with high- and low-stem cell percentages, indicating that SCFR did not serve as the sole predictor of proliferative ability in these cultures.

RNA sequencing (RNA-seq) genome-wide transcript analysis performed using RNA from SUM-159 cell cultures with high and low stem-like cell percentages identified 1884 differentially expressed genes (DEGs, FDR value < 0.05) in the high *versus* low stem cell groups, consisting of 1059 upregulated and 825 downregulated genes (Supporting Information Fig. S7). Pathway analysis and gene ontology clustering analyses performed with these DEGs identified multiple signaling pathways (Fig. 5C) that revealed differential upregulation in the high *vs*. low stem cell groups, including the mitogen-activated protein kinase (MAPK), NF-*κ*B, and JAK–STAT pathways, as well as several pathways related to cancer metabolism. Gene ontology clustering (Fig. 5D) revealed the stem-cell-enriched groups differentially expressed genes related to cell proliferation, differentiation, regulation of apoptosis, metabolism, and secretion. Changes in select genes from functionally relevant pathways were also verified and quantified by qPCR (Fig. 5E).

Activating mutations in the canonical RAS/MAPK pathway occur at a relatively low rate (2%–10%) in breast cancer^42^. However, RAS/MAPK pathway activity is linked to breast cancer progress^43^^,^^44^ and maybe dysregulated by overexpression of upstream receptor tyrosine kinases such as human epidermal growth factor receptors 1 and 2 (aka HER1 and HER2), as commonly observed in breast cancer^45^. Since EGF is a MAPK regulatory target that plays a significant role in breast cancer progression and exhibited the largest changes in our RNA-seq and secretome analyses, we evaluated whether MAPK pathway activation and EGF stimulation were sufficient to induce stem cells to transdifferentiate or replicate from *in vivo* breast cancer cell transplants. EGF treatment induced similar proliferation of SUM159 cells natively expressing wild-type KRAS (WT) or overexpressing an activated KRAS mutant allele (G12V) during *in vitro* culture but increased the stem-like cell fraction only in G12V cell cultures (Fig. 5F), indicating that both EGF exposure and elevated KRAS activity were required for stem cell proliferation and/or transdifferentiation. However, a more complex response was observed when female BALB/c Nude mice were injected in the fourth mammary fat pad with equal amounts (10^5^) of cells from these four cultures and monitored for 7 weeks by bioluminescent imaging to assess tumor growth (Fig. 5G). In this experiment, the mice injected with G12V + EGF culture cells exhibited greater tumor area than the injected with G12V or WT + EGF culture cells only at 7 weeks post-injection (Fig. 5H), although there was a marked difference in the rate of total fluorescence signal development among these groups (G12V + KRAS > KRAS > EGF), indicating G12V and EGF treatment had synergistic effects to promote tumor growth, with the combination of both inducing a rapid increase in cancer cell expansion.

## 4 Discussion

This study characterizes the performance of a robust and versatile microfluidic platform for automated single live-cell separation and culture and highly reproducible *in situ* serial analysis of cell biomarkers and secretion patterns over extended on-chip culture intervals. Single-cell capture events occur through the uniform distribution of fluid and cells through the chip microchannels that minimize shear stress and cell damage. Continuous perfusion of the chip spoke microchannels from the hub well prevents cross-contamination. At the same time, this radial separation of approach eliminates the potential interference of upstream cultures on downstream cultures since a single culture is established per channel^46^. This also permits the continuous supply of nutrients and removal of toxic metabolites, essential to maintaining high cell survival and growth rates^47^ and facilitating long-term on-chip culture of single-cell isolates. This design enables high-throughput analysis of biomarker expression on individual cells to facilitate the identification of clonal phenotype diversity, minimizes sample consumption, permits collection of concentrated conditioned media, and streamlines operational procedures.

Single-cell analyses are needed to reveal the importance of tumor heterogeneity^48^, ^49^, ^50^, which can complicate cancer classification, diagnosis, and treatment^51^. Our microfluidic chip design allows automatic multi-step operations to permit continuous multidimensional analysis of single-cell heterogeneity. We used this method for long-term monitoring of single-cell cultures derived from six breast cancer cell lines. Single-cell proliferative capacity and survival rates were associated with high SCFR secretion levels. Comparative secretome analyses of cells with varying proliferative capacities, phenotypes, and proportion of stem-like cells identified a set of cytokines that may serve as potential therapeutic markers. This device can thus serve as a platform to analyze cell heterogeneity and screen for biomarkers associated with specific cell subtypes and treatment responses. Further, unlike other chip-based methods where pre-determined surface modifications limit the range of analyses possible for a single-cell culture^52^^,^^53^, our method collects concentrated supernatants that can be analyzed using off-chip assays.

Malignant transformation is consistently associated with disruptions in vital regulatory mechanisms. However, cancer cell populations can maintain stable phenotypic characteristics for extended periods, including subpopulations with distinct surface biomarker profiles that can remain unchanged through multiple divisions in cell culture. Single-cell proliferation and phenotyping assays on our chip detected a consistent equilibrium in the cell subpopulations derived from these single-cell cultures, which did not depend on the parental cell’s phenotypic subtype. These findings indicate that stochastic effects, not lineage effects, determine phenotypic equilibria within cancer cell populations to influence tumor dynamics and provide confirmatory evidence that individual stem-like and non-stem-like cells can interconvert^54^^,^^55^. We also observed that chemotherapeutic drug exposure could shift this balance to increase the proportion of stem-like cells with tumor-seeding capacity.

This platform can effectively characterize cells that exhibit increased conversion to stem-like phenotypes following chemotherapeutic treatment and the cell pathways associated with this enhanced phenotypic conversion rate. In this study, chemokine treatment enhances the proportion of stem-like cells and secretome and RNA results from cell populations that exhibited a high level of stem-cell expansion after single-cell culture revealed increases in signaling pathways and processes associated with cell survival, proliferation, differentiation, and metabolism, including MAPK/RAS signaling and EGF secretion. MAPK signaling plays an essential role in breast cancer progression and has been targeted by numerous therapeutics designed to inhibit tumor growth^45^^,^^56^. However, our on-chip indicates that KRAS activation and EGF exposure are required to promote conversion to a stem-like phenotype and induce the most robust tumor response when used to seed a mouse breast cancer model.

Our current device offers several advantages over current methods used for single-cell analysis (Supporting Information Table S3). However, several approaches could be used to improve its performance. Refinements to its geometry and/or serially repeating the trapping process could increase its throughput and culture area. Future studies could use this platform to address several critical remaining questions about the single-cell interconversion process, including secretory kinetic information in single-cell clones and a detailed analysis of the dynamics of the transition process. This platform can also be applied to analyze the phenotype changes in other cell types in the presence or absence of specific environmental or genetic perturbations, including immune cells and non-malignant or precancerous cell lines. On-chip evaluation of the multidimensional processes that regulate specific cell behaviors in these single-cell cultures will be fundamental to understanding stochastic processes and mechanisms involved in specific phenotypic transitions.

## 5 Conclusions

In summary, this work developed a novel strategy to accomplish a series of single-cell operations to characterize the proliferative potential, phenotype, and secretion of single cells. Our findings suggest that the single cells maintained phenotypic equilibrium based on surface markers, and chemotherapy drugs caused phenotypic plasticity among them. Combined with secretome analysis, we screened many cytokines correlated with single-cell growth, phenotype, and stemness. The platform has the potential to achieve more complex research on single-cell heterogeneity.

## Author contributions

Zhun Lin: Writing – original draft, Methodology, Investigation. Siping Liang: Methodology. Zhe Pu: Methodology. Zhengyu Zou: Methodology. Luxuan He: Methodology. Christopher J. Lyon: Writing – review & editing, Supervision. Yuanqing Zhang: Writing – review & editing, Supervision, Conceptualization. Tony Y. Hu: Writing – review & editing, Supervision. Minhao Wu: Writing – review & editing, Supervision.

## Conflicts of interest

The authors have no conflicts of interest to declare.

## Appendix A Supporting information

The following is the Supporting Information to this article:
