# Supplementary material for: Phenotypic plasticity and secretory heterogeneity in subpopulations derived from single cancer cell
Source: Acta Pharm Sin B. 2025 Mar 15;15(5):2723–35. doi: 10.1016/j.apsb.2025.02.039 (PMC12144973; doi:10.1016/j.apsb.2025.02.039)
Supplement: Multimedia component 1 [file mmc1.docx]

**Supporting Information for**

**Original article**

**Phenotypic plasticity and secretory heterogeneity in subpopulations**

**derived from single cancer cell**

**Zhun Lin^a^ , Siping Liang^b^ , Zhe Pu^a^ , Zhengyu Zou^b^ , Luxuan He^a^ , Christopher J.**

**Lyon^c^ , Yuanqing Zhang^a,*^,Tony Y. Hu^c,*^, Minhao Wu^b,*^**

^a^*School of Pharmaceutical Sciences, Sun Yat-sen University, Guangzhou 510006,*

*China*

^b^*Zhongshan School of Medicine, Sun Yat-sen University, Guangzhou 510080, China*

^c^*Center of Cellular and Molecular Diagnosis, Tulane University School of Medicine,*

*New Orleans, LA 70112, USA*

Received 18 September 2024; received in revised form 12 December 2024; accepted

5 January 2025

*Corresponding authors.

E-mail addresses: wuminhao@mail.sysu.edu.cn (Minhao Wu), TonyHu@tulane.edu

(Tony Y. Hu), zhangyq65@mail.sysu.edu.cn (Yuanqing Zhang).


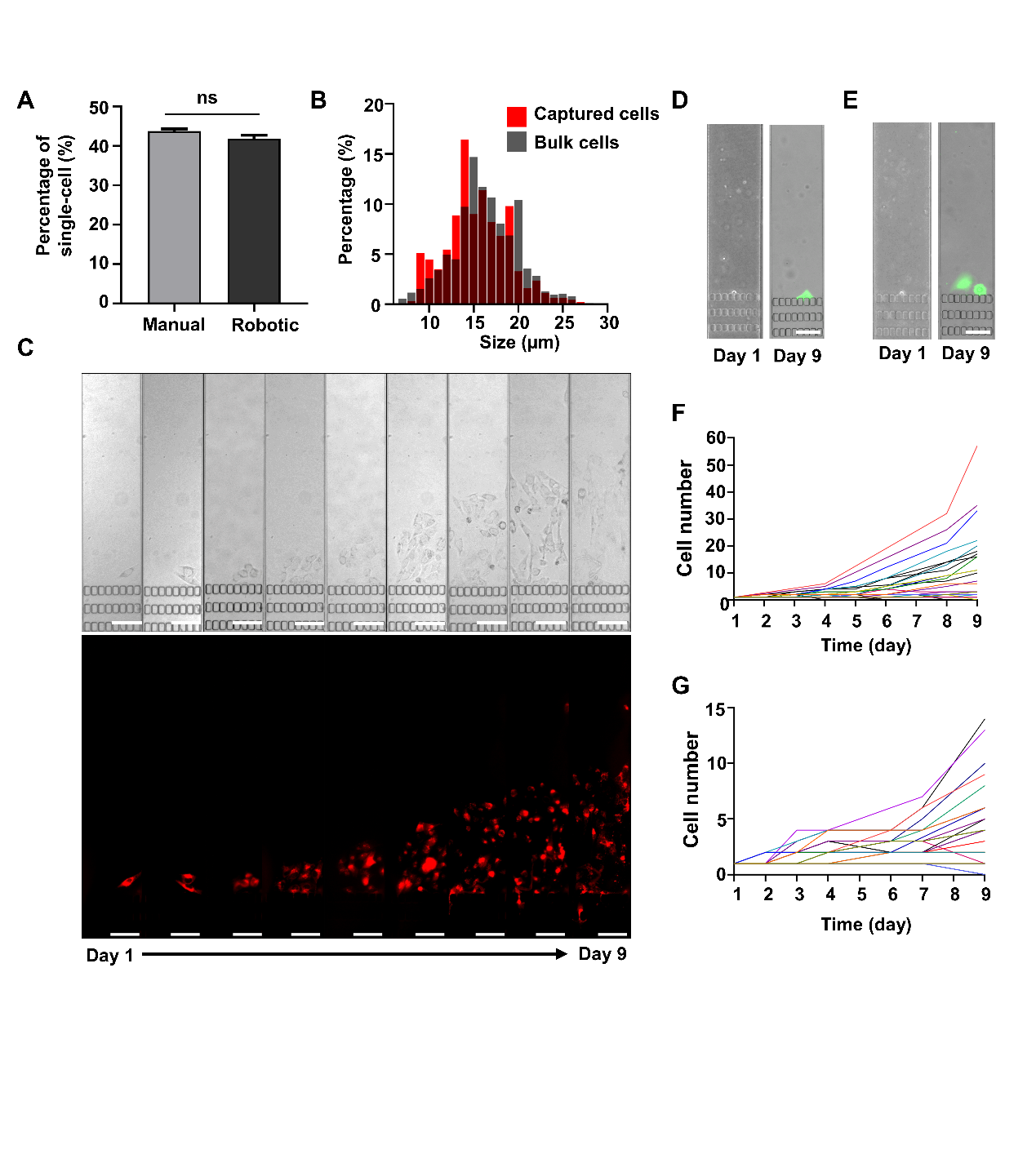


Figure S1 Single-cell capture and on-chip proliferative heterogeneity. (A) No differences were observed when the microchip was manually or robotically loaded to generate single-cell assays. Differences were analyzed by a two-tailed Mann-Whitney test (ns, not significant) (n=3/group). (B) Comparison of the size distribution of MCF-7 cells captured on the microchip capture and present in the source population. (C) Images of cell proliferation over time in a representative single-cell culture well, as viewed with visible light (top) and by fluorescence illumination of Cell Tracker-stained cells (bottom) (scale bars: 100 μm). (D, E) Images of Day 9 single-cell MCF-7 cultures that (D) failed to proliferate or (E) stagnated after one cell division (scale bar: 100 μm). (F, G) Cell numbers in subpopulations derived from single-cell (F) MDA-MB-231 and (G) MCF-7 cultures over a nine-day culture interval.


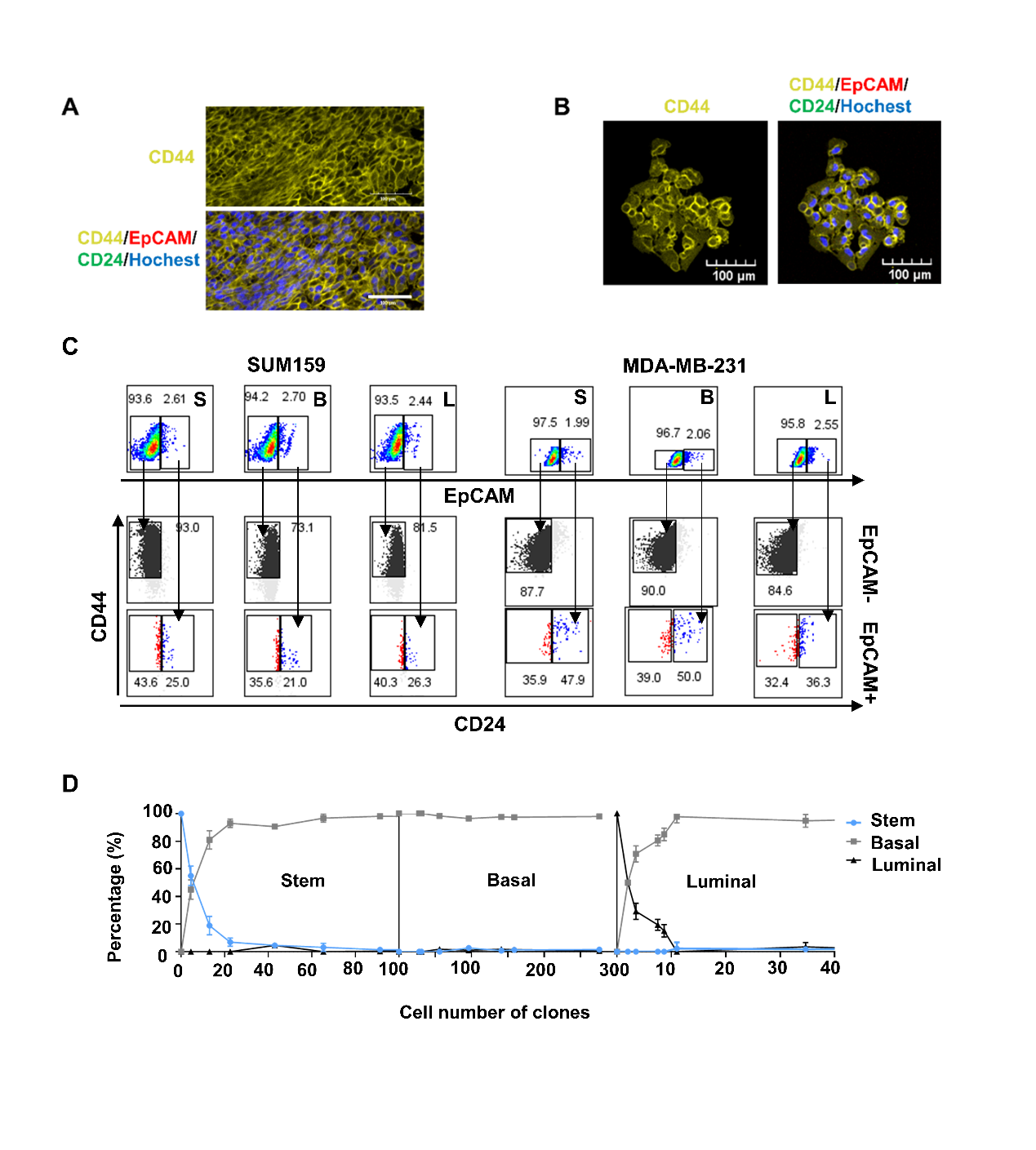


**Figure S2** Characterization of cell subtypes in the single-cell clones and their source cultures. (A, B) Confocal micrographs indicating CD44, CD24, and EpCAM expression detected by immunofluorescence staining in (A) a parental SUM-159 cell population and (B) a single-cell culture derived from these cells (scale bar: 100 µm). (C) Flow cytometry analysis of S/B/L-type cell subpopulations present in standard cultures of the SUM-159 and MDA-MB-231 cell lines. (D) Changes in the percentage of S-, B-, and L-type cells in single cell cultures established with the indicated cell subtypes.


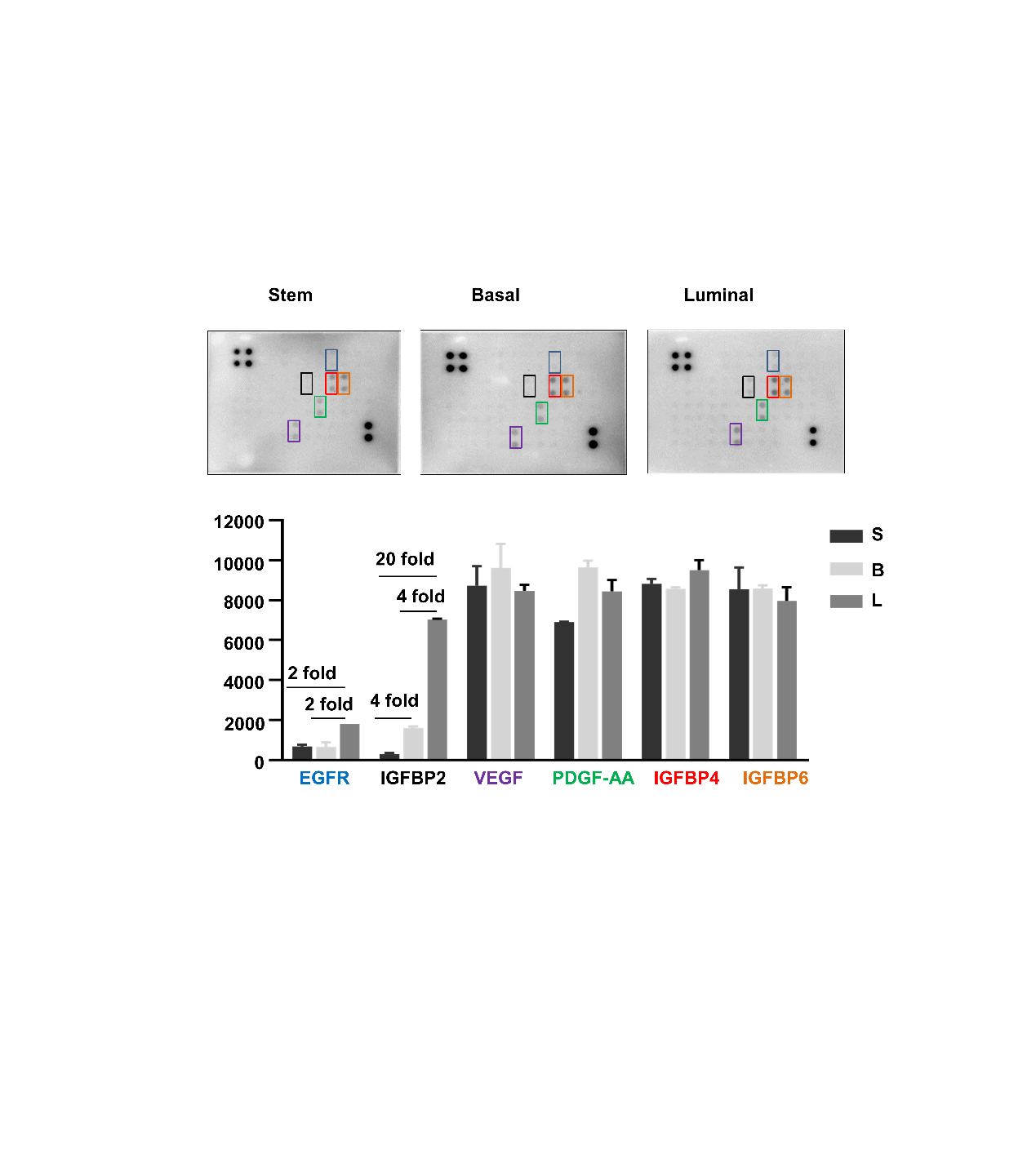
Figure S3 Comparison of growth factors in CMs from subpopulations derived from single-cell cultures of S-, B-, and L-type cells, indicating factors differentially detected in CM of these subtype cultures when analyzed on an antibody array (indicated by colored boxes), as presented as the mean and standard deviation densitometric values detected at duplicate spots on the array. Stem: S; Basal: B; Luminal: L.


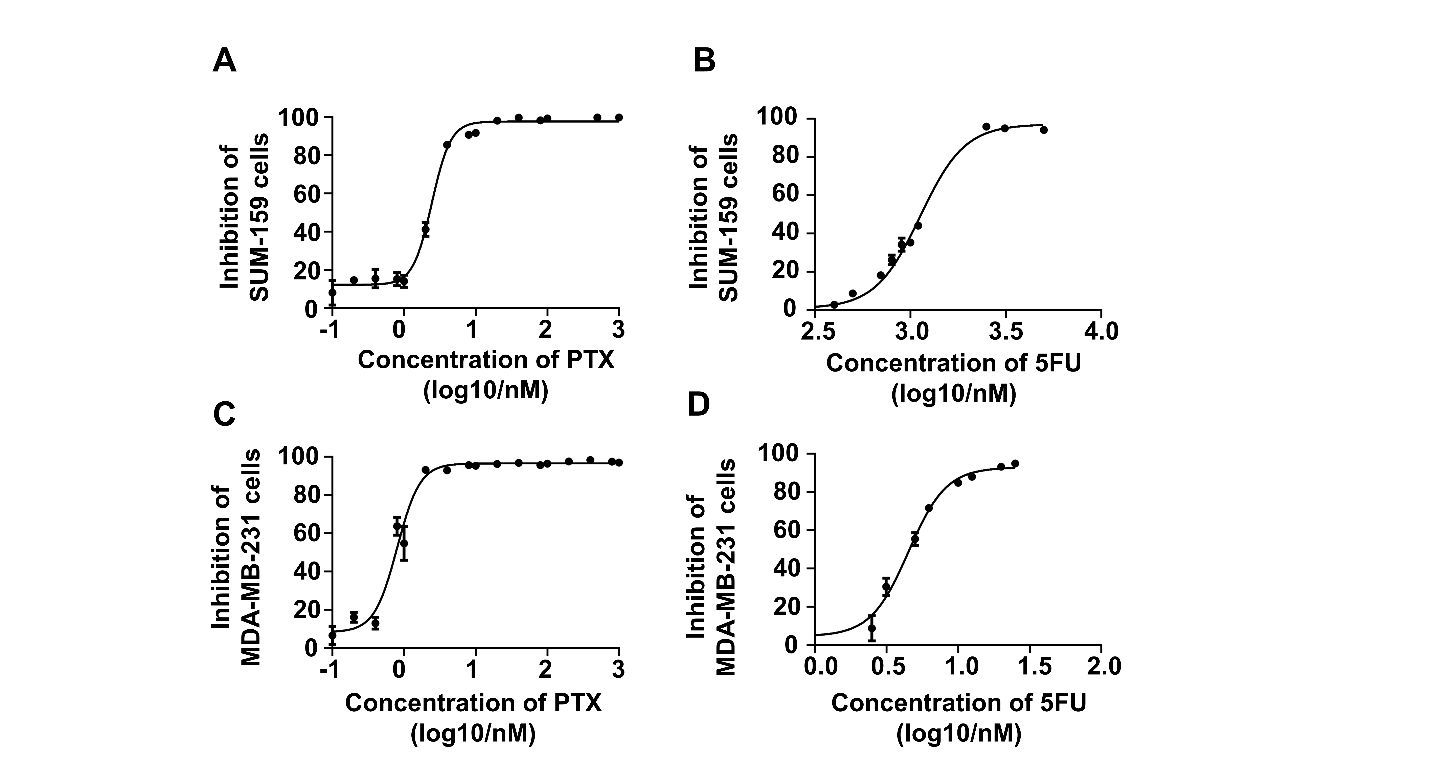


Figure S4 CCK8 assay results indicating the dose-dependent inhibitory responses detected in SUM-159 cell cultures treated with (A) PTX and (B) 5-FU and MAD-MB-231 cell cultures treated with (C) PTX and (D) 5-FU.


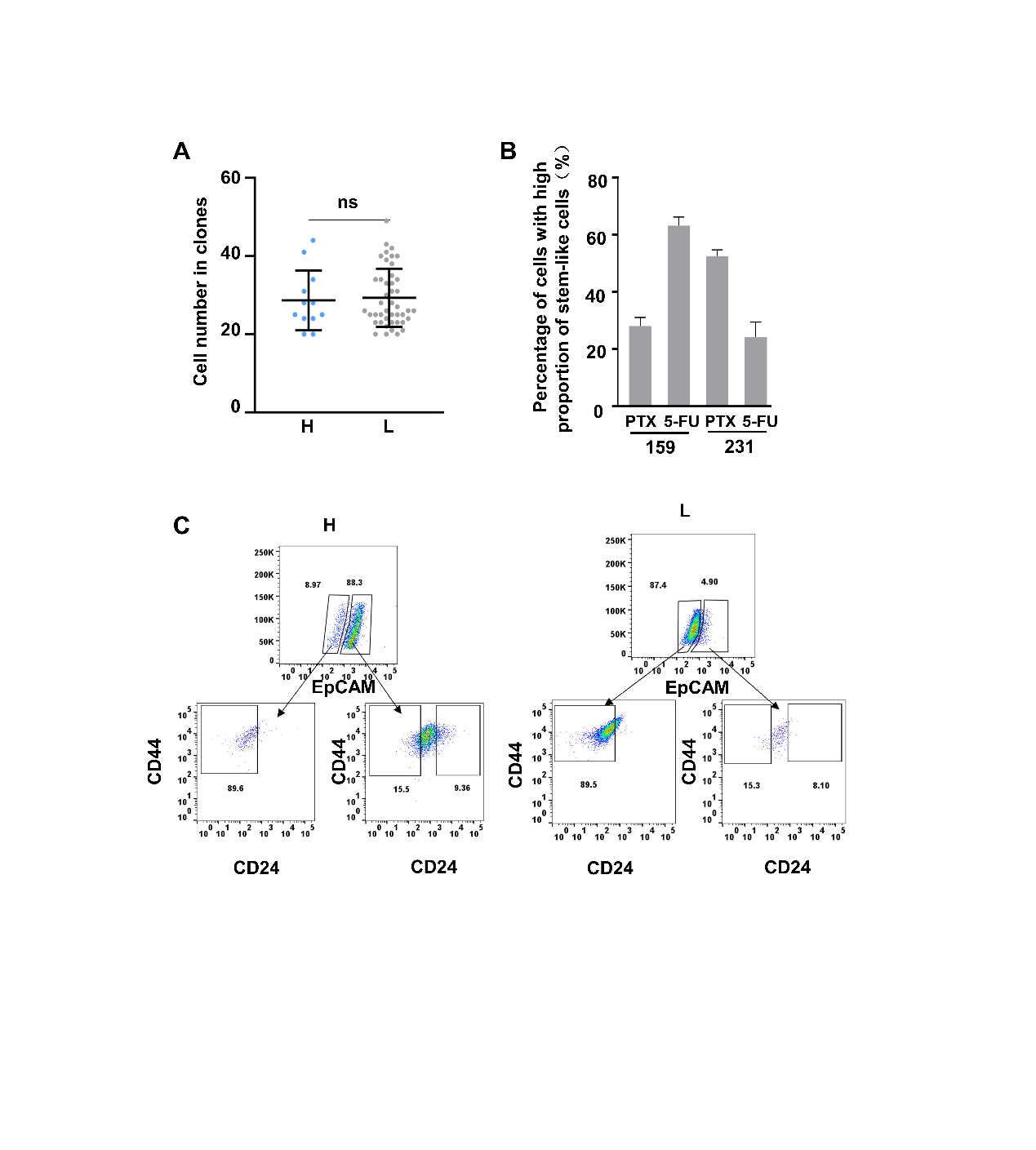


Figure S5 Characteristics of single-cell-derived populations with high and low percentages of stem-like cells. (A) Cell numbers in clones of PTX-treated SUM-159 single-cell cultures that produced high (H) and low (L) percentages of stem-like cells, where differences were analyzed by a two-tailed Mann-Whitney test (ns, not significant). (B) Percentage of cells exhibiting a high proportion of stem-like cells in single-cell cultures of SUM-159 (159) and MDA-MB-231 (231) cells after treatment with the indicated drugs (n=3/group). (C) Flow cytometry analysis of populations with high and low fractions of stem-like cells derived from single-cell SUM-159 cultures after PTX treatment.


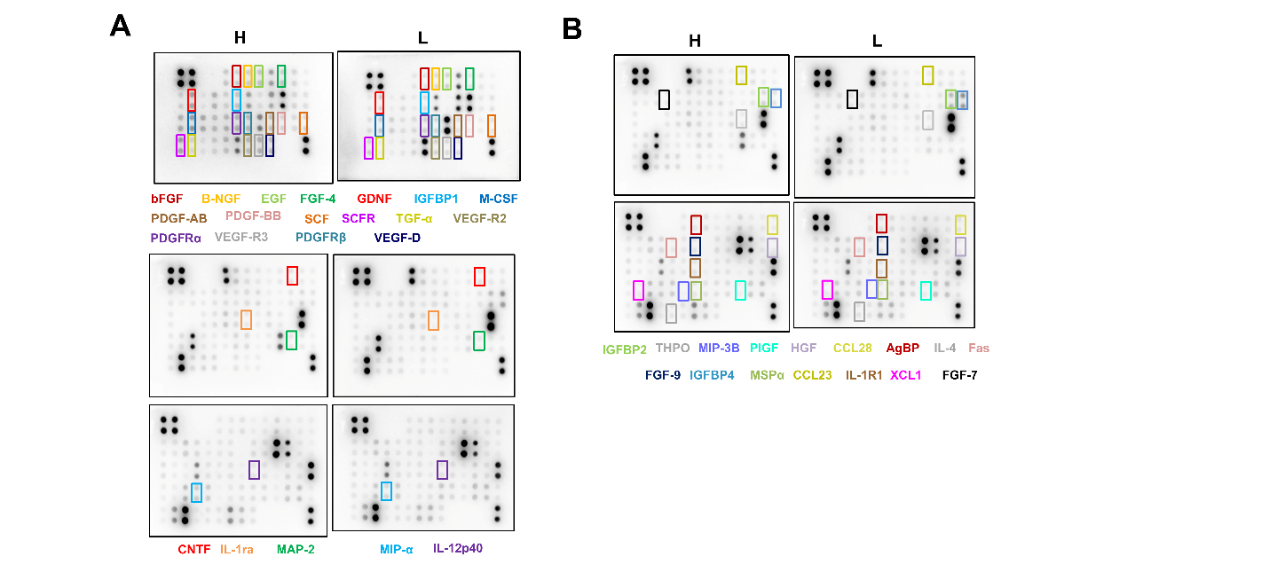


Figure S6 Factors (A) increased and (B) decreased in CMs isolated from cell populations derived from single-cell SUM-159 cultures that exhibited high (H) *vs*. low (L) proportion of stem-like phenotypes, where colored boxes indicate factors with altered expression between these two groups.

**
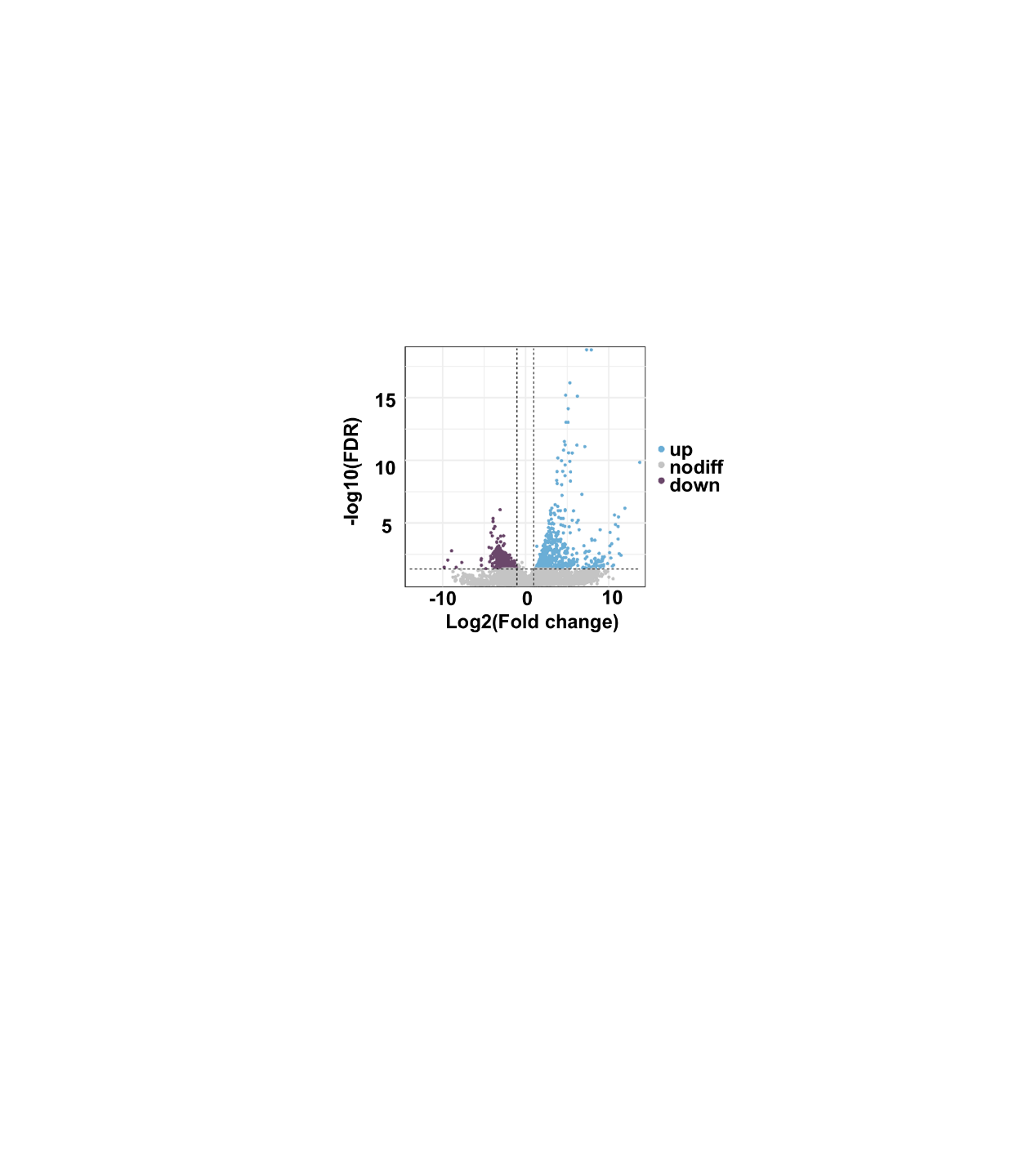
**

**Figure S7** Volcano plot of differentially expressed gene between high and low fractions of stem-like cells. The blue dots represent up-regulation genes, and the purple dots represent down-regulation genes.

**Table S1** The specific primer sequences

|  | Forward primer sequence (5’-3’) | Reverse primer sequence (5’-3’) |
| --- | --- | --- |
| IL1RAP | ACACTTCTGTGGTGTGTAGTGA | TGGTGTCTAGTCCCCAGTCAT |
| TGFBR1 | ACGGCGTTACAGTGTTTCTG | GCACATACAAACGGCCTATCTC |
| STK4 | CCTCCCACATTCCGAAAACCA | GCACTCCTGACAAATGGGTG |
| KRAS | ACAGAGAGTGGAGGATGCTTT | TTTCACACAGCCAGGAGTCTT |
| CHUK | GGCTTCGGGAACGTCTGTC | TTTGGTACTTAGCTCTAGGCGA |
| SOS2 | CCGCAGCCTTACGAGTTCTTC | GGATGCACTTGTTCCTGAACC |
| PRKACB | CCATGCACGGTTCTATGCAG | GTCTGTGACCTGGATATAGCCTT |
| RASA1 | ACTTGACAGAACGATAGCAGAAG | GCCTCCGATCACTCTCTCTTA |
| TAOK1 | ATGCCATCAACTAACAGAGCAG | CGCACATCTCGTGCAAAATAC |
| *β*-actin | ACTCTTCCAGCCTTCCTTCC | CGTACAGGTCTTTGCGGATG |

**Table S2** The inhibitory concentrations (IC) values for SUM-159 and MDA-MB-231 population cell viability of paclitaxel (PTX) and 5-fluorouracil (5-FU)

| IC value | SUM-159 | | MDA-MB-231 | |
| --- | --- | --- | --- | --- |
|  | PTX (nM) | 5-FU (μM) | PTX (nM) | 5-FU (μM） |
| 90 | 4.760±0.691 | 2.032±0.413 | 1.798±0.292 | 8.761±1.369 |
| 70 | 3.161±0.265 | 1.414±0.165 | 1.095±0.099 | 5.822±0.613 |
| 50 | 2.445±0.149 | 1.126±0.092 | 0.803±0.074 | 4.505±0.460 |
| 30 | 1.891±0.148 | 0.897±0.099 | 0.588±0.083 | 3.486±0.453 |

**Table S3 The comparison of this work with reported single-cell culture methods.**

| **Method** | **Single-cell rate** | **Time of culture** | **Area of culture** | **Number of chambers** | **Function** |
| --- | --- | --- | --- | --- | --- |
| Iso-osmotic perfusion microfluidic cell culture array (*Nat Methods*  . **2011** May 22;8(7):581-6.) | 10-30% | 72 hours | 160 μm ×160 μm ×100 μm | 1,600 | Culture, immunostaining, conditioned media collection, cell retrieval |
| Alginate microparticles generator (*Adv Healthc Mater*  . **2015** Aug 5;4(11):1628-33.) | 25% | 15 days | 10 - 50 µm in diameter | Limitless | Culture, retrieval |
| Single-cell phenotyping assay  (Our work) | 44% | 9 days | 200 μm ×1000 μm ×25 μm | 120 | Culture, immunostaining, conditioned media collection, retrieval |
